# The metastasis inducer CCN1 (CYR61) activates the fatty acid synthase (FASN)-driven lipogenic phenotype in breast cancer cells

**DOI:** 10.18632/oncoscience.314

**Published:** 2016-07-22

**Authors:** Javier A. Menendez, Luciano Vellon, Ingrid Espinoza, Ruth Lupu

**Affiliations:** ^1^ ProCURE (Program Against Cancer Therapeutic Resistance), Metabolism and Cancer Group, Catalan Institute of Oncology, Girona, Catalonia, Spain; ^2^ Girona Biomedical Research Institute (IDIBGI), Girona, Spain; ^3^ IBYME, CONICET-Laboratorio de Immunohematología, Buenos Aires, Argentina; ^4^ Cancer Institute, University of Mississippi, Jackson, MS, USA; ^5^ Department of Preventive Medicine, University of Mississippi, Jackson, MS, USA; ^6^ Mayo Clinic, Department of Laboratory Medicine and Pathology, Division of Experimental Pathology, Rochester, MN, USA; ^7^ Mayo Clinic Cancer Center, Rochester, MN, USA

**Keywords:** CCN1, CYR61, fatty acid synthase, breast cancer, metastasis

## Abstract

The angiogenic inducer CCN1 (Cysteine-rich 61, CYR61) is differentially activated in metastatic breast carcinomas. However, little is known about the precise mechanisms that underlie the pro-metastatic actions of CCN1. Here, we investigated the impact of CCN1 expression on fatty acid synthase (FASN), a metabolic oncogene thought to provide cancer cells with proliferative and survival advantages. Forced expression of CCN1 in MCF-7 cells robustly up-regulated FASN protein expression and also significantly increased *FASN* gene promoter activity 2- to 3-fold, whereas deletion of the sterol response element-binding protein (SREBP) binding site in the *FASN* promoter completely abrogated CCN1-driven transcriptional activation. Pharmacological blockade of MAPK or PI-3'K activation similarly prevented the ability of CCN1 to induce *FASN* gene activation. Pharmacological inhibition of FASN activity with the mycotoxin cerulenin or the small compound C75 reversed CCN1-induced acquisition of estrogen independence and resistance to hormone therapies such as tamoxifen and fulvestrant in anchorage-independent growth assays. This study uncovers FASNdependent endogenous lipogenesis as a new mechanism controlling the metastatic phenotype promoted by CCN1. Because estrogen independence and progression to a metastatic phenotype are hallmarks of therapeutic resistance and mortality in breast cancer, this previously unrecognized CCN1-driven lipogenic phenotype represents a novel metabolic target to clinically manage metastatic disease progression.

## INTRODUCTION

CCN1, also known as CYR61, is an extracellular matrix-associated protein [1, 2] belonging to the Cysteine rich 61/Connective tissue growth factor/Nephroblastoma overexpressed (CCN) gene-family of survival and angiogenic regulators, which includes CCN2 (CTGF), CCN3 (NOV), CCN4 (WISP-1), CCN5 (WISP-2), and CCN6 (WISP-3) [3–10]. Whereas all CCN proteins have been shown to mediate functions as diverse as cell proliferation, migration, adhesion, differentiation, and extracellular matrix formation, CCN1 is unique among CCN proteins because of its ability to regulate more complex processes, such as angiogenesis and tumorigenesis [11–19].

CCN1 is overexpressed in about 30% of triplenegative breast carcinomas (TNBC) [14, 15]. Although TNBC is clearly defined based upon immunohistological criteria (estrogen receptor-negative, progesterone receptor-negative, and HER2-negative), it remains a biologically heterogeneous disease encompassing a number of intrinsic molecular subtypes, most frequently basal-like and claudin-low [20–22]. Resistance of TNBC to standard therapies drastically limits the available options for previously treated patients with metastatic TNBC and there is currently no preferred standard chemotherapy. Not surprisingly, TNBC accounts for a disproportionate number of metastatic disease cases and breast cancer deaths [23–25].

CCN1 expression strongly correlates with known markers for invasiveness, and associates with the ability of breast cancer cells to invade *in vitro* and metastasize *in vivo* [14–16]. Indeed, a significant correlation exists between elevated levels of CCN1 and an advanced stage of the primary tumor and lymph node involvement at the time of surgery [14, 26, [27]. We previously demonstrated that CCN1 is an important regulator of the vascular compartment in breast cancer, with strong stimulatory effects on tumor neovascularization that ultimately promotes the progression and metastatic dissemination of breast carcinomas [13, 15, [16]. Moreover, CCN1 expression inversely correlates with estrogen receptor expression, response to estradiol, and sensitivity to antiestrogen and taxanes-based therapies [18, 28, [29]. Whereas CCN1 overexpression-driven estrogen independence and progression to a metastatic phenotype are hallmarks of therapeutic resistance and mortality in breast cancer [30–33], the precise mechanisms by which CCN1 promotes more aggressive breast cancer metastatic phenotypes remain unknown.

Most cancer cells do not use circulating fatty acids for energy, but instead endogenously synthesize them *de novo* for growth and survival in the unfavorable microenvironment of solid tumors or metastases prior to angiogenesis. Indeed, a fatty acid synthase (FASN)- driven “lipogenic state”, by conferring growth and survival advantages, and cross-talking with established cancer-controlling signaling pathways, appears to necessarily accompany the natural history of most human cancers [34–38]. Accordingly, the oncogenic nature of FASN overexpression confers tumor aggressiveness and poor prognosis in various human cancers. However, the role of aberrant expression of key lipogenic enzymes such as FASN on metastasis-prone phenotypes remains enigmatic [39]. In this work, we aimed to examine the influence of the metastasis inducer CCN1 on the expression of cancer cell-associated FASN as well as the role of FASN activity on some of the key metastatic features acquired by CCN1-overexpressing breast cancer cells.

## RESULTS

### CCN1 up-regulates FASN protein expression in breast cancer cells

**Figure 1:**
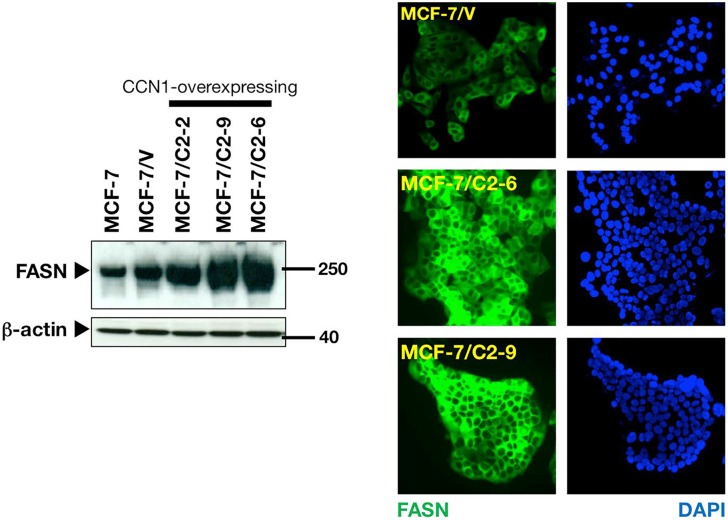
CCN1-overexpressing MCF-7 breast cancer cells up-regulate FASN protein expression *Left.* Cells were cultured in IMEM-5% FBS to 75% confluence, washed with PBS and solubilized in lysis buffer. Aliquots (10 μg) of 14 000 × g supernatants were fractioned by NuPAGE, transferred to nitrocellulose membranes, probed with an anti-FASN monoclonal antibody, and then re-probed with an anti-β-actin antibody. Representative immunoblotting analysis is shown (n = 3). *Right.* Cells were fixed and labeled with an anti- FASN monoclonal antibody. Cells were extensively washed, and FASN protein was detected by indirect immunofluorescence using FITCconjugated anti-mouse IgG. Cells were counterstained with DAPI and examined with a Zeiss fluorescent microscope. Representative FASN immunostaining analysis is shown. Similar results were obtained in three independent experiments.

To evaluate the impact of CCN1 on FASN expression, we utilized a cellular model of CCN1 overexpression developed in our laboratory. Estrogendependent MCF-7 breast cancer cells, which naturally express very low to undetectable levels of CCN1, were transfected with full-length CCN1 cDNA [16, 28] and stable cell clones were selected with zeocin. Analysis of expanded cell clones by western blotting showed that FASN protein expression was markedly higher in the CCN1-expressing clones C2-2, C2-9 and C2-6 [28] than in empty vector-transfected cells (MCF-7/V) and parental MCF-7 cells (Figure 1, *left panel*). Indeed, CCN1 overexpression was sufficient to increase steady-state FASN protein to levels similar to those found in SKBR3 cells (data not shown), a naturally occurring FASNoverexpressing breast cancer cell line.

To assess whether the accumulation of FASN protein was uniform in cells overexpressing CCN1, the cellular patterns of FASN expression were assessed by microscopy (Figure 1, *right panel*). Although direct quantitative interpretation of indirect immunofluorescence is not possible, it was evident that cytoplasmic accumulation of FASN was higher in CCN1-overexpressing clones than in MCF-7/V cells. The difference in FASN intensity between CCN1-overexpressing clones and MCF-7/V cells was manifest and highly reproducible. These results reveal that CCN1 overexpression leads to up-regulation of cancerassociated FASN.

**Figure 2:**
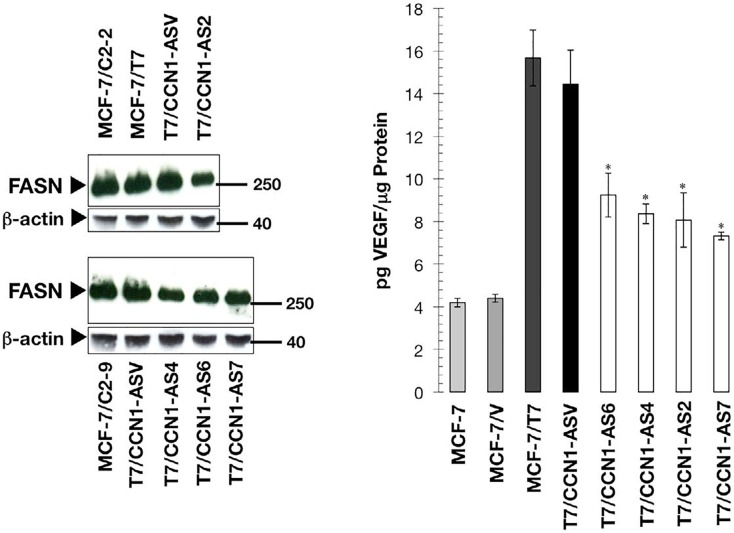
Blockade of CCN1 down-regulates FASN and VEGF in HRG-overexpressing breast cancer cells *Left.* Cells were cultured in IMEM-5% FBS to 75% confluence, washed with PBS and solubilized in lysis buffer. Aliquots (10 μg) of 14 000 × g supernatants were fractioned by NuPAGE, transferred to nitrocellulose membranes, probed with an anti-FASN monoclonal antibody, and then re-probed with an anti-β-actin antibody. Representative immunoblotting analysis is shown (n = 3). *Right.* Cells were serum-starved overnight and then cultured in 0.1% FBS-IMEM for 48 h. Culture supernatants were collected to determine the level of VEGF_165_ by ELISA, which was normalized to the amount of protein in collected cell extracts. Secretion levels of VEGF_165_ in AS-CCN1 clones were compared to those in HRG/CCN1-overexpressing MCF-7/T7 cells. *P < 0.005.

To confirm that the ability of CCN1 to regulate FASN was independent of the up-stream CCN1 activator HRG, we evaluated FASN protein expression after blockade of *CCN1* gene expression in MCF-7 cells engineered to overexpress HRG (MCF/T7 cells) [28]. FASN expression in MCF-7/T7 cells was similar to that found in the CCN1-overexpressing clones C2-2 and C2-9 (Figure 2, *left*) Moreover, FASN expression was lower in cell lysates from antisense CCN1 clones T7/CCN1-AS2, T7/CCN1-AS4, T7/CCN1-AS6, and T7/CCN1-AS7 than in MCF-7/T7 cells (Figure 2, *left*). Additionally, in HRG-overexpressing T7/CCN1- AS cells with intact HRG Æ HER2/3 signaling in the absence of CCN1 expression, there was a striking correlation between the decrease in FASN expression and the reduced secretion of the angiogenic factor VEGF_165_ (Figure 2, *right*).

These findings reveal that up-regulation of a FASNdependent lipogenic phenotype constitutes part of the prometastatic program resulting from CCN1 overexpression in breast cancer.

### CCN1 stimulates *FASN* gene expression in breast cancer cells

**Figure 3:**
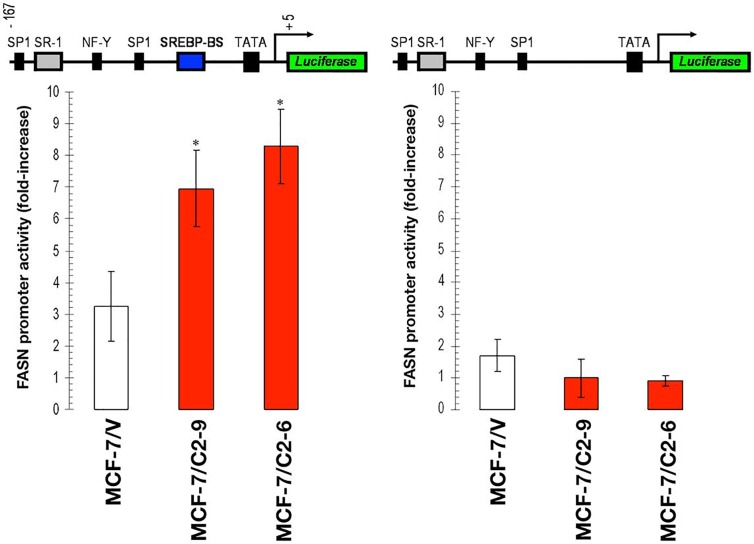
CCN1 overexpression activates the *FASN* gene promoter in a SREBP-dependent manner Cells were transiently transfected with a plasmid containing a luciferase reporter gene driven by a 178-bp *FASN* gene promoter fragment harboring a SREBPbinding site, flanked by auxiliary NF-Y and Sp-1 sites (*left*) or with a similar construct in which the SREBP domain was deleted (*right*). Luciferase activity was expressed as relative (*fold*) change in transcriptional activities of *FASN* promoter-transfected cells after normalization to pRL-CMV activity. Each experimental value represents the mean fold increase (*columns*) ± S. D. (*bars*) from at least three separate experiments in which triplicate wells were measured. Luciferase activity in CCN1-overexpressing clones was compared to that in MCF-7/V control cells. *P < 0.005.

To determine whether CCN1 overexpression affected *FASN* gene transcription, C2-6 and C2-9 clones and MCF-7/V control cells were transfected with a reporter construct containing a 178-bp *FASN* promoter fragment harboring all the elements necessary for highlevel expression of FASN, including a complex SREBPbinding site [40–42]. CCN1 overexpression significantly increased luciferase activity 2- to 3-fold relative to baseline levels observed in MCF-7/V cells (Figure 3, *left*). To address whether the SREBP-binding sites present in the proximal region mediated the effects of CCN1 on *FASN* promoter activation, cells were transiently transfected with a *FASN* promoter in which the SREBP binding region was deleted (FASNdelSRE) [40–42]. CCN1-driven stimulation of the *FASN* promoter was completely abolished in the absence of the SREBP binding region (Figure 3, *right*).

Collectively, these results show that CCN1-driven stimulation of FASN expression seems to take place, at least in part, at the transcriptional level and is mediated by *cis*-acting elements such as the SREBP-binding sites present in the proximal *FASN* gene promoter.

### CCN1 stimulates *FASN* gene expression *via* PI- 3K and MAPK signaling cascades

Having previously demonstrated that CCN1 overexpression activates PI-3K/AKT and MEK1/MEK2/ERK1/ERK2 signaling in MCF-7 cells [16, 28], we postulated that CCN1-enhanced FASN expression might result from activation of these signaling cascades in CCN1-overexpressing cells.

**Figure 4:**
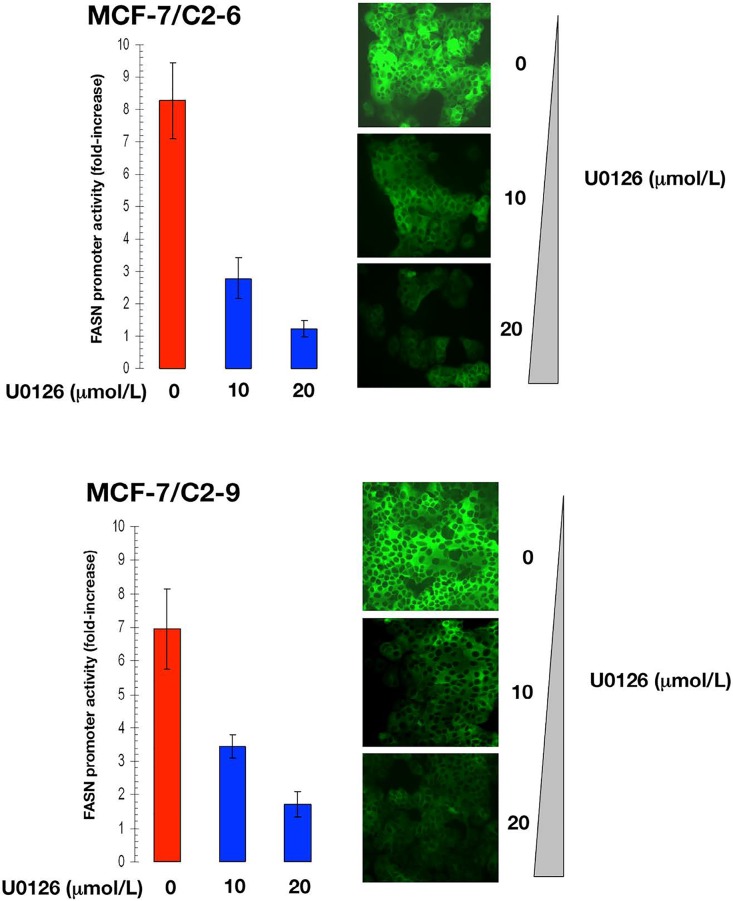
CCN1 overexpression up-regulates FASN *via* activation of the ERK/MAPK pathway Cells were transiently transfected with a plasmid containing a luciferase reporter gene driven by a 178-bp *FASN* gene promoter fragment harboring a SREBPbinding site, flanked by auxiliary NF-Y and Sp-1 sites. The next day, cells were treated with graded concentrations of U0126. After 24 h, cells were lysed and luciferase activity was measured. Luciferase activity was expressed as relative (*fold*) change in transcriptional activities of *FASN* promoter-transfected cells in response to U0126 treatments after normalization to pRL-CMV activity. Each experimental value represents the mean fold increase (*columns*) ± S. D. (*bars*) from at least three separate experiments in which triplicate wells were measured. Luciferase activity in U0126-treated cells was compared to that in vehicle-treated control cells. *P < 0.005.

**Figure 5:**
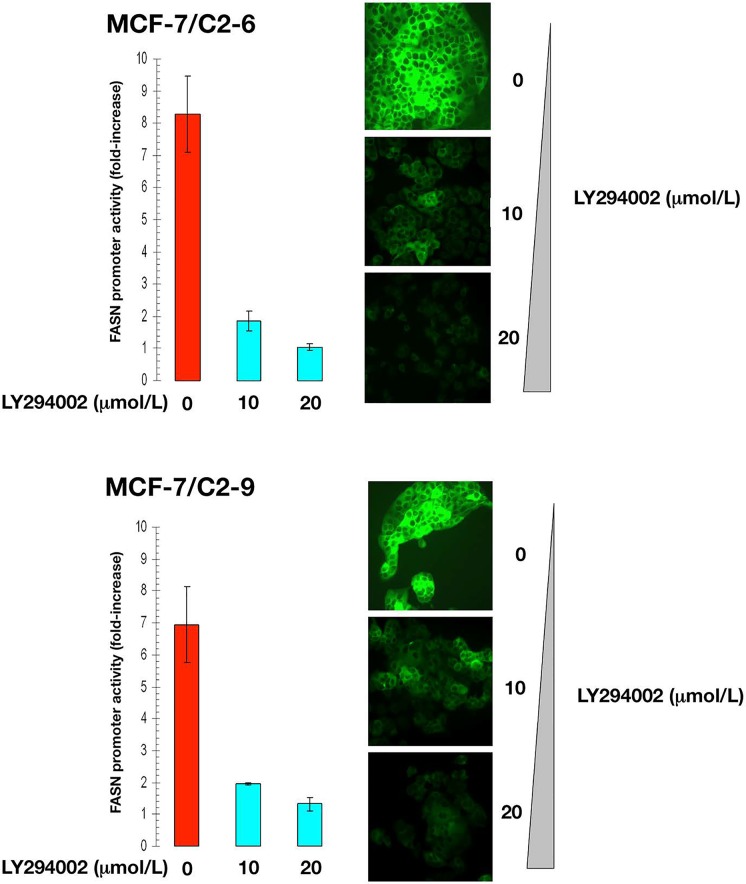
CCN1 overexpression up-regulates FASN via activation of the PI-3K pathway Cells were transiently transfected with a plasmid containing a luciferase reporter gene driven by a 178-bp *FASN* gene promoter fragment harboring a SREBP-binding site, flanked by auxiliary NF-Y and Sp-1 sites. The next day, cells were treated with graded concentrations of LY294002. After 24 h, cells were lysed and luciferase activity was measured. Luciferase activity was expressed as relative (*fold*) change in transcriptional activities of *FASN* promoter-transfected cells in response to LY294002 treatments after normalization to pRL-CMV activity. Each experimental value represents the mean fold increase (*columns*) ± S. D. (*bars*) from at least three separate experiments in which triplicate wells were measured. Luciferase activity in LY294002-treated cells was compared to that in vehicle-treated control cells.^*^P < 0.005.

Treatment of C2-9 and C2-6 clones with nontoxic concentrations of U0126, a potent inhibitor of the MEK/ERK MAPK pathway, dose-dependently decreased the stimulatory effects of CCN1 on the activity of the *FASN* promoter (Figure 4). Furthermore, treatment with nontoxic concentrations of LY294002, a potent cell permeable inhibitor of PI-3K, drastically suppressed the activity of the *FASN* promoter in C2-9 and C2-6 cells (Figure 5). Additionally, U0126- and LY294002-mediated downregulation of FASN protein expression was detected by immunocytochemistry (Figure 4 and 5). Immunoblotting procedures confirmed the ability of U0126 and LY294002 to dose-dependently decrease FASN protein expression in *CCN1*-overexpressing breast cancer cells (data not shown).

Altogether, these results provide evidence for a CCN1-triggered regulatory cascade that actively links MAPK and PI-3K transduction upstream of the *FASN* gene, with SREBP-dependent transcriptional regulation of the *FASN* promoter.

### Pharmacological inhibition of FASN activity reverses CCN1-promoted estrogen independence and anti-estrogen resistance

Because MCF-7 cells overexpressing CCN1 are estradiol (E_2_)-independent and acquire an antiestrogen resistant phenotype [16, 18], a prevalent clinical occurrence in breast cancer progression [30–33], we next evaluated whether exacerbated FASN activity might serve as part of the molecular program by which CCN1 promotes an aggressive breast cancer phenotype. To do this, we measured anchorage-independent growth as an *in vitro* metric of tumorigenicity.

**Figure 6:**
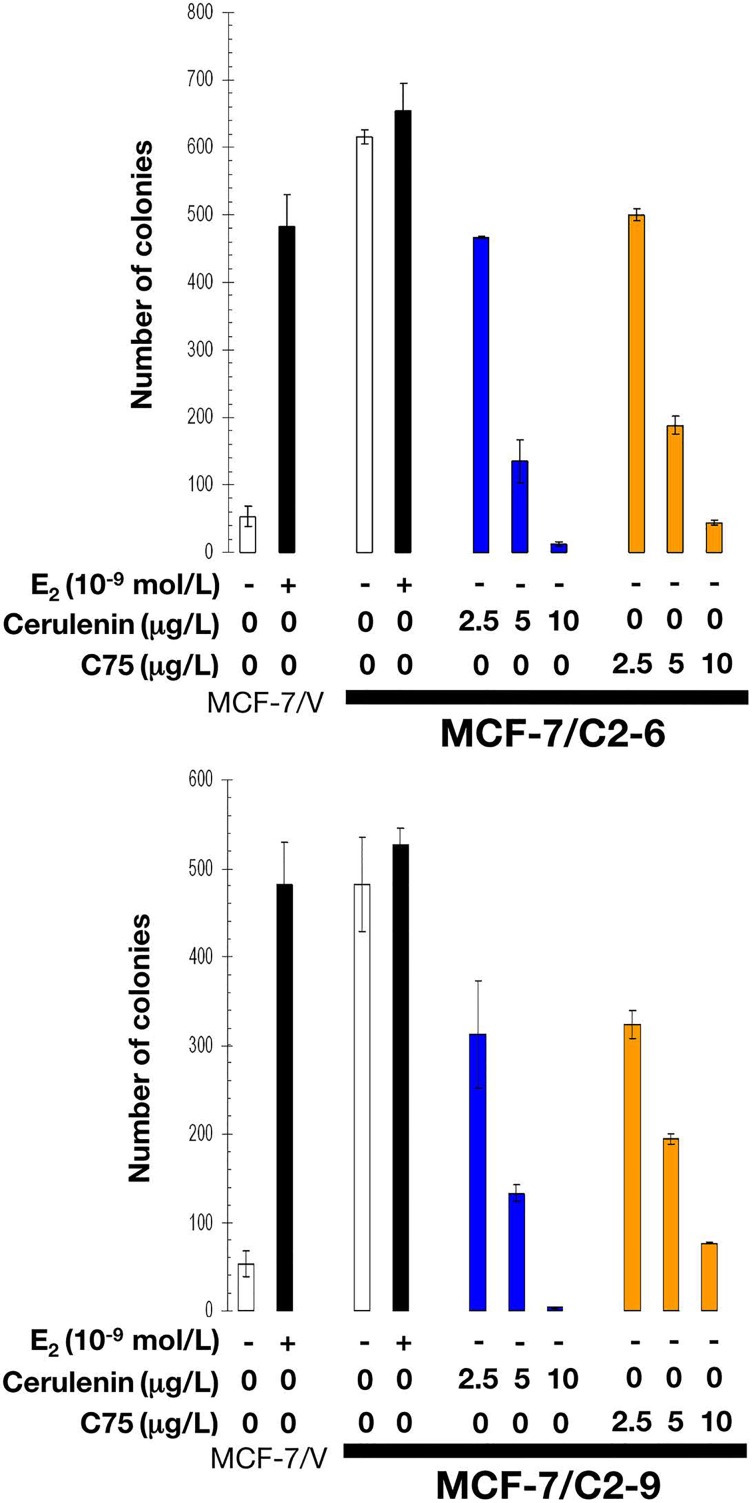
Pharmacological inhibition of FASN activity impedes estrogen-independent cell growth of CCN1- overexpressing breast cancer cells E_2_-depleted cells were plated in soft agarose containing E_2_ (10^−9^ mol/L), cerulenin (2.5, 5, and 10 μg/mL), C75 (2.5, 5, and 10 μg/ml), or ethanol (*v/v*) or DMSO (*v/v*) vehicle only for 7-10 days. Colony formation (≥ 50 μm) was assessed using a colony counter. Each experimental value represents the mean colony number (*columns*) ± S. D. (*bars*) from at least three separate experiments in which triplicate dishes were counted.

Neither wild-type (not shown) nor empty vectortransfected MCF-7/V cells formed colonies in soft-agar assays in the absence of estradiol. By contrast, C2-9 and C2-6 clones showed a strong anchorage-independent capacity to form colonies in the absence of estradiol (Figure 6). This estradiol-independent colony forming ability was dramatically suppressed in a dose-dependent manner with the mycotoxin cerulenin or its semi-synthetic derivative C75 (α-methylene-γ-butyrolactone) (Figure 6), which are two widely used small-molecule FASN inhibitors [43–45].

**Figure 7:**
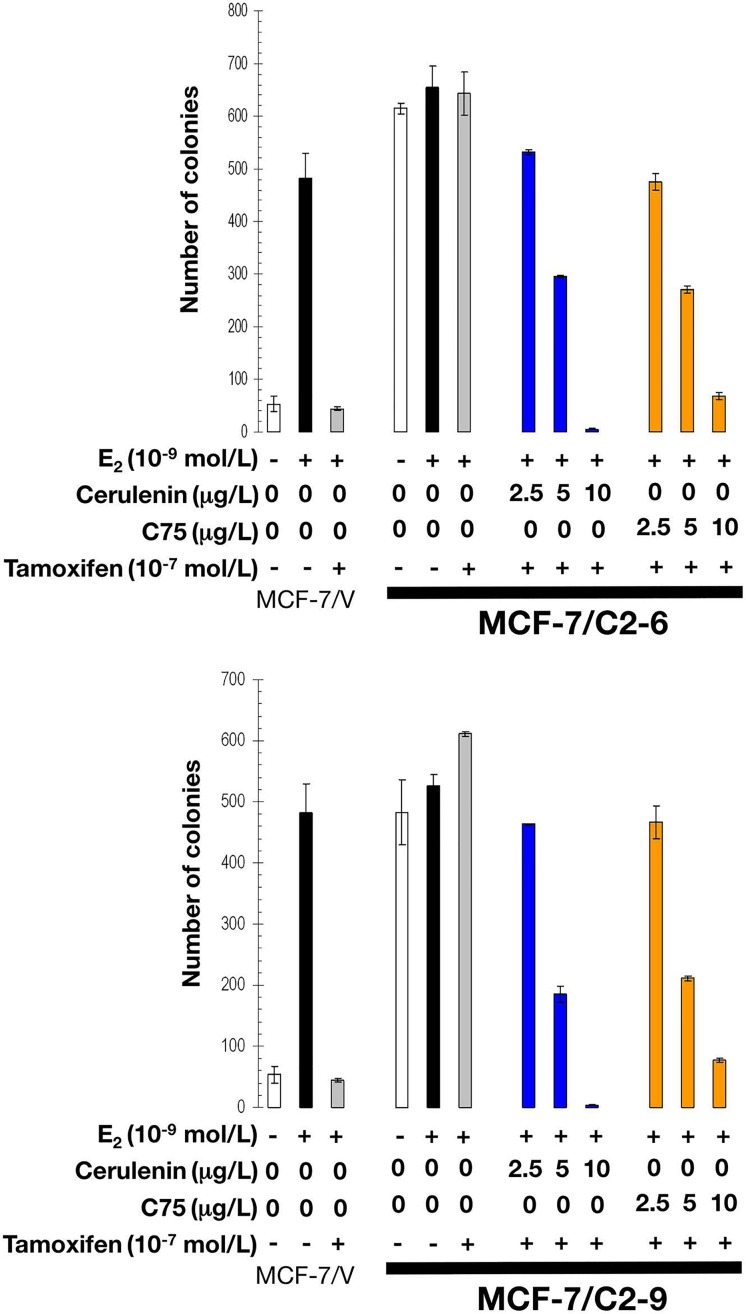
Pharmacological inhibition of FASN activity reverses tamoxifen resistance of CCN1-overexpressing breast cancer cells E_2_-depleted cells were plated in soft agarose containing E2 (10^−9^ mol/L), tamoxifen (10^−7^ mol/L), cerulenin (2.5, 5, and 10 μg/mL), C75 (2.5, 5, and 10 μg/ml), their combinations, or ethanol (*v/v*) or DMSO (*v/v*) vehicle only for 7-10 days. Colony formation (≥ 50 μm) was assessed using a colony counter. Each experimental value represents the mean colony number (*columns*) ± S. D. (*bars*) from at least three separate experiments in which triplicate dishes were counted.

**Figure 8:**
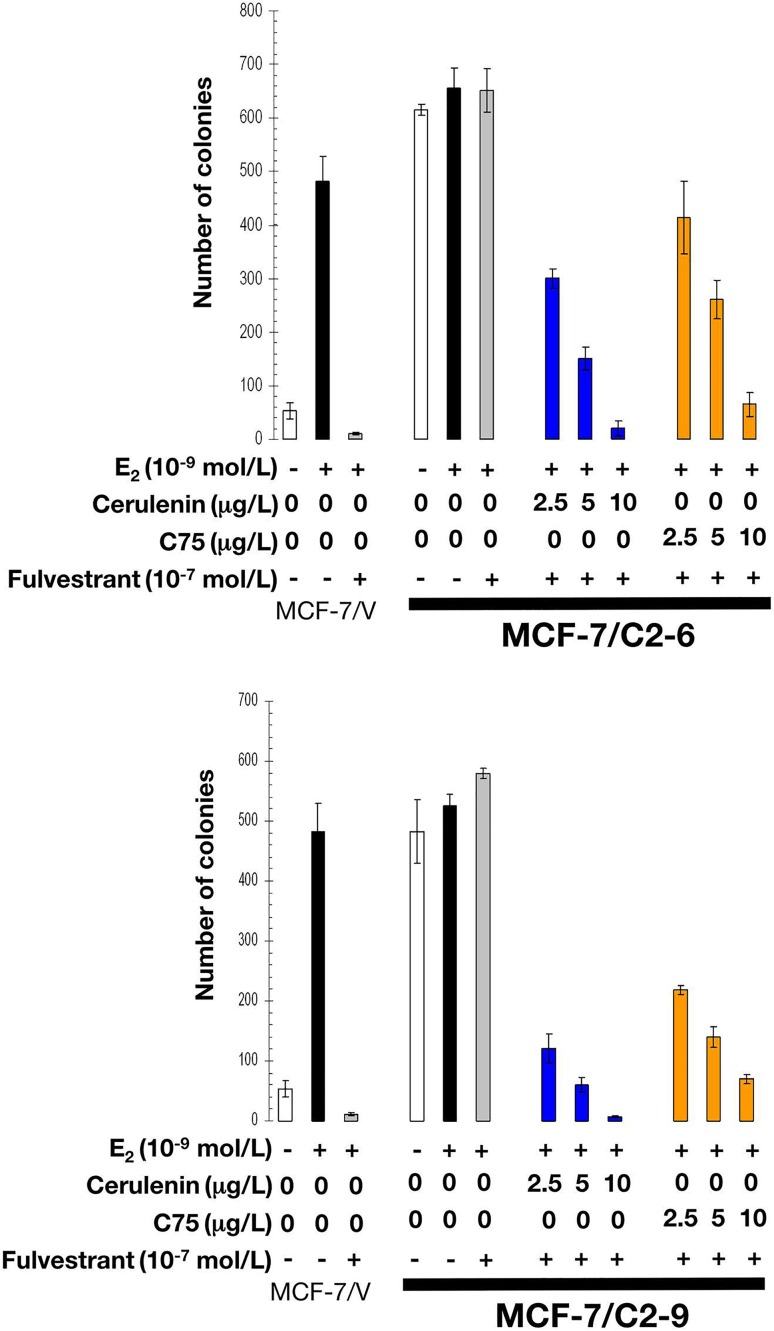
Pharmacological inhibition of FASN activity reverses fulvestrant resistance of CCN1-overexpressing breast cancer cells E_2_-depleted cells were plated in soft agarose containing E_2_ (10^−9^ mol/L), fulvestrant (10^−7^ mol/L), cerulenin (2.5, 5, and 10 μg/mL), C75 (2.5, 5, and 10 μg/ml), their combinations, or ethanol (*v/v*) or DMSO (*v/v*) vehicle only for 7-10 days. Colony formation (≥ 50 μm) was assessed using a colony counter. Each experimental value represents the mean colony number (*columns*) ± S. D. (*bars*) from at least three separate experiments in which triplicate dishes were counted.

As expected, estradiol exposure robustly induced anchorage-independent growth of MCF-7/V cells, which was prevented by the antiestrogens tamoxifen and fulvestrant. Whereas estradiol treatment modestly stimulated anchorage-independent colony formation of C2-9 and C2-6 cells, neither tamoxifen nor fulvestrant prevented the strong colony forming capacity of these cells in the presence of estradiol (Figures 7 and 8). Strikingly, the FASN inhibitors cerulenin and C75 dose-dependently suppressed anchorage-independent colony formation when used in combination with tamoxifen or fulvestrant. Furthermore, the ability of FASN inhibitors to restore the sensitivity of CCN1/CYR61-overexpressing cells to antiestrogens was extremely effective with the pure antiestrogen fulvestrant (Figure 8), which antagonizes the hormone-dependent activation of estrogen receptors but lacks the mixed antagonist/agonist effects of tamoxifen.

Collectively, these findings reveal that FASN-driven endogenous lipogenesis is part of the molecular signaling cascade through which CCN1 overexpression promotes progression to a metastatic phenotype *via* acquisition of estrogen independence and antiestrogens resistance.

### Pharmacological inhibition of FASN activity induces higher levels of cellular damage in CCN1-overexpressing breast cancer cells

**Figure 9:**
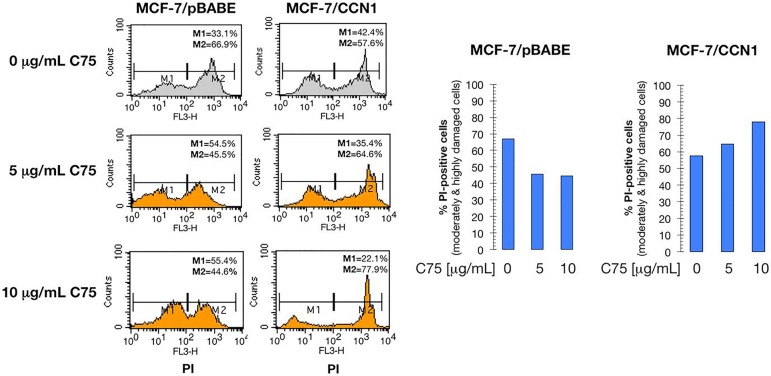
CCN1 overexpression exacerbates breast cancer cell sensitivity to FASN inhibition Serum-starved CCN1- negative MCF-7/pBABE control cells and CCN1-overexpressing MCF-7/CCN1 cells were treated with C75 (0, 5, and 10 μg/mL) for 2 days. Left panels show the distribution of M1 (PI-negative, undamaged) and M2 (PI-positive, moderately and highly damaged) cell populations in the absence/presence of C75. Right panels show the effect of different doses of C75 on undamaged/damaged cell populations using a bar-graph representation.

To definitely explore the notion that FASN-driven endogenous lipogenesis might constitute an attractive therapeutic target for eliminating CCN1-overexpressing breast cancer cells, we employed a flow cytometric fluorescence-based method to discriminate damaged/dead from viable cells in immunofluorescently labeled populations using propidium iodide (PI) as a dyeexclusion viability probe. We employed MCF-7 cells stably transduced with pBABE-CCN1 or pBABE (empty control) retroviral vectors to confirm that CCN1-driven FASN overexpression was not due to non-physiological CCN1 expression levels in selected clones. When PI uptake was plotted against FSC (forward scattering) to monitor the response to graded concentrations of C75, vector control MCF-7/pBABE cells exhibited a subdued response with no clear cut dose dependence. Conversely, a gradual loss of viability was clearly observed for MCF- 7/CCN1 cells as the C75 concentration was increased gradually up to 10 μg/mL (Figure 9).

Because an increase in PI uptake can be viewed as a reliable indicator of cell injury severity, our results suggest that pharmacological blockade of FASN activity causes significantly increased amounts of cellular damage in CCN1-overexpressing when compared with CCN1-negative breast cancer cells.

## DISCUSSION

We reveal for the first time that FASN, a key enzyme for endogenous fatty acid biogenesis whose overexpression is associated with more aggressive subsets of breast carcinomas and poorer clinical outcomes, constitutes part of the cellular signaling cascade orchestrated by CCN1 to drive breast cancer cell growth, angiogenesis and metastatic progression.

In the present study, we show that CCN1 significantly stimulates *FASN* gene transcription and FASN protein accumulation in breast cancer cells. In agreement with earlier reports on the up-regulatory effects of androgens, progestins, growth factors, growth factor receptors, and hypoxia on *FASN* gene activity, CCN1- stimulated FASN expression seems to involve activation of the SREBP pathway in breast cancer cells [40–42, 46–49]. Accordingly, CCN1 overexpression stimulates the transcriptional activity of a short and well-defined *FASN* promoter harboring a complex SREBP-binding site, whereas its specific deletion completely abolishes the stimulatory effects of CCN1. Moreover, the use of various inhibitors of different signal transduction pathways further reveals that, upstream of SREBP, the effects of CCN1 on *FASN* gene expression are complex and likely involve the activation of multiple molecular transducers. Consequently, pharmacological blockade of MEK1/MEK2 and PI-3K activity blunted the up-regulatory effects of CCN1 overexpression on *FASN* gene activity and FASN protein accumulation. The results of quantitative real-time RT-PCR confirmed CCN1-driven induction of FASN through MAPK and PI-3K-mediated pathways, as measured by luciferase reporter and immunoblotting expression analyses (data not shown). These findings strongly suggest that CCN1 overexpression triggers a signaling cascade that, up-stream of the *FASN* gene, links the ERK1/ERK2 MAPK and PI-3K transduction pathways with SREBP.

That FASN expression becomes activated in response to high levels of the angiogenic inducer CCN1 together with the finding that the status of FASN expression parallels CCN1-regulated secretion of the archetypical angiogenic factor VEGF_165_ might counterintuitively suggest that FASN signaling plays an active role in CCN1-driven breast cancer angiogenesis, a crucial step for metastasis. However, earlier studies indeed revealed that cancer cell-associated FASN can actively regulate tumor vasculature through altering the profile of secreted angiogenic factors including VEGF165, and regulation of their bioavailability [50]. Although it remains to be determined whether FASN-driven lipogenesis might directly participate in the increased neovascularization that we previously reported in tumors formed by HRG- and CCN1-overexpressing breast cancer cells, attenuation of FASN-dependent endogenous lipogenesis has been shown to abolish the establishment of metastatic colonies without significantly affecting the growth of the primary tumor [51]. Remarkably, FASNdriven enhanced lipid synthesis has been shown to be instrumental for the metabolic shift associated with a drastic increase in metastatic dissemination following cancer adaptation and resistance to antiangiogenic treatments [52]. Because pharmacological and genetic ablation of FASN overcomes tumor regrowth and metastasis after antiangiogenic therapy withdrawal [52], it might be worthwhile to evaluate whether the CCN1 Æ FASN angiogenesis/lipogenesis axis is responsible for the resistance to antiangiogenic therapies [53, 54].

The expression of genes with pro-angiogenic ontology including VEGF and, consequently, excessive angiogenesis, have been shown to accompany the acquisition of estrogen independence and resistance to anti-estrogens [55–59]. CCN1 overexpression can molecularly substitute for estradiol and foster hormoneindependent growth and resistance to tamoxifen and fulvestrant. Thus, our findings showing that FASN inhibition blocks anchorage-independent growth of CCN1-overexpressing breast cancer cells in the absence of estradiol while restoring their responsiveness to antiestrogens, strongly suggests that CCN1-driven FASN overexpression might be part of the proangiogenic phenotype which, if recapitulated *in vivo*, may promote tumor progression. Given that the potential of a cancer cell to proliferate and colonize a soft agar environment is a surrogate marker of aggressive behavior *in vitro* and indicative of invasion, therapeutic resistance and metastatic dissemination, our data identify FASN as a potential therapeutic target to improve the efficacy of angiogenesis-related development of resistance to antiestrogens.

In summary, we present the first evidence that overexpression of the metastatic/angiogenic inducer CCN1 is sufficient to enable a metabolic shift involving FASNdriven enhanced lipid synthesis in a cell-autonomous manner. CCN1-induced preactivation of a metabolic infrastructure providing proliferative and survival benefits to cancer cells under stressful microenvironmental conditions might constitute an efficient preadaptive strategy through which CCN1-overexpressing cellular states would obtain an advantage in hostile milieus, such as those accompanying metastatic dissemination and colonization [60]. Because much shorter median time from relapse to death remains an urgent unmet need for metastatic TNBC patients, the discovery of FASN as a novel molecular feature directly involved in CCN1- promoted breast cancer metastatic progression might accelerate the incorporation of new generations of FASN inhibitors [61] in the management of metastatic TNBC.

## MATERIALS AND METHODS

### Materials

Improved Minimal Essential Medium (IMEM) and phenol red-free IMEM were from Biofluids (Rockville, MD). Fetal bovine serum (FBS) was from Nova-Tech Inc. (Grand Island, NY). Dextran-coated charcoaltreated bovine serum (CCS) was from BioSource International (Camarillo, CA). Estradiol (E2) was from Sigma-Chemical Co. (St. Louis, MO). Fulvestrant (ICI- 182,780) was a gift from AstraZeneca. LY29400, a specific inhibitor of the p110 catalytic subunit of PI-3K, and the MEK1/MEK2 inhibitor U0126 were purchased from Calbiochem (San Diego, CA), dissolved in DMSO, and stored as 10 mmol/L stock solutions in the dark at −20°C until use. Cerulenin and C75 (racemic) were purchased from Sigma-Chemical Co. and Alexis Biochemicals (San Diego, CA), respectively. Cerulenin and C75 were dissolved in DMSO and stored in the dark as a stock solution (25 mg/ml) at −20°C until use. For treatments, LY294002, U0126, cerulenin, and C75 were freshly prepared from stock solutions and diluted with growth medium. Control cells were cultured in medium containing the same concentration of DMSO (*v/v*) as the experimental cultures with cerulenin or C75.

The primary antibody (Ab) for FASN immunoblotting was a mouse IgG_1_ FAS monoclonal Ab (clone 23) from BD Biosciences Pharmingen (San Diego, CA). An anti-β-actin goat polyclonal antibody was purchased from Santa Cruz Biotechnology (Santa Cruz, CA).

### Cell culture

MCF-7 breast cancer cells were obtained from the American Type Culture Collection (ATCC) and were grown in IMEM supplemented with 5% (*v/v*) FBS and 2 mmol/L L-glutamine at 37°C in a humidified atmosphere of 95% air and 5% CO_2_.

MCF-7 cells were engineered to overexpress CCN1 as described [16, 28]. Briefly, cells were electroporated with the eukaryotic expression vector pcDNA3.1/zeocin(−) containing the full-length cDNA of human CCN1, or with an empty vector as a negative control. Stably transfected cells were selected with zeocin (200 μg/ml) for 2 weeks. Because CCN1 mRNA and protein expression and cellular behavior was similar in most of the clones, a representative vector (V3–2) and three clones (C2-2, C2-6 and C2-9) were chosen for further analysis.

To block endogenous CCN1 expression, fulllength CCN1 cDNA was cloned in an antisense direction into pcDNA3.1/zeocin(−) and transfected into HRG-overexpressing MCF-7/T7 cells, a breast cancer progression model developed in our laboratory by transfecting full-length HRG-β2 cDNA into non-metastatic MCF-7 cells [62, 63]. Several CCN1 antisense (T7/CCN1- AS) clones were isolated and the presence of antisense CCN1 mRNA was confirmed by RNAse protection assay. We also generated multiple clones of vector-transfected MCF-7/T7 (T7/AC-V) cells, and all behaved similarly to the wild-type MCF-7/T7 clone. Four T7/CYR61-AS clones (T7/CCN1-AS2, T7/CCN1-AS4, T7/CCN1-AS6, and T7/CCN1-AS7) and one vector clone (T7/AC-V1) were chosen for further analysis. CCN1-overexpressing MCF-7 clones and CCN1-AS MCF-/T7 clones were grown as described, except than 200 μg/ml of zeocin was added to the culture medium.

A pool MCF-7/CCN1 cell line was generated by transducing MCF-7 cells with a retroviral vector (pBABE) containing the full-length cDNA for CCN1. Cell lines were selected with puromycin (3 μg/mL) (MCF-7/CCN1), and a control cell line was generated in parallel, under similar conditions using the empty retroviral vector (MCF- 7/pBABE) alone.

### FASN promoter luciferase assays

Cells were transfected with FuGENE 6 (Roche Biochemicals, Indianapolis, IN). Overnight serumstarved cells seeded into 24-well plates (5 × 10^4^ cells/well) were transfected for 18 h in low-serum (0.1% FBS) medium with 300 ng/well of the pGL3-luciferase (Promega, Madison, WI) vector containing a luciferase reporter gene cloned downstream of an intact (FASN wtSREBP-BS-Luc) 178-bp FASN promoter fragment, and pRL-CMV (30 ng/well), which was used to correct for transfection efficiency. Transfected cells were washed and incubated with/without graded concentrations of U0126 or LY294002. Approximately 24 h after treatments, luciferase activity from cell extracts was detected using the Luciferase Assay System (Promega, Madison, WI) according to the protocol specified by the manufacturer using a VICTOR2^™^ 1420 Multilabel Counter (Perkin Elmer^™^). The level of activation in FASN promotertransfected cells was determined after normalization to the luciferase activity in cells co-transfected with equivalent amounts of the empty pGL3-luciferase vector lacking the FAS promoter (Ø-Luc) and the internal control plasmid pRL-CMV, which was taken as 1.0-fold. This control value was used to calculate the fold change in transcriptional activities of FASN promoter- transfected cells in response to either CNN1/CYR61 overexpression or U0126/LY294002 treatments after normalization to pRL-CMV activity.

### Immunoblotting analysis of FASN

Cells were washed twice with PBS and lysed in a buffer (20 mmol/L Tris (pH 7.5), 150 mmol/L NaCl, 1 mmol/L EDTA, 1 mmol/L EGTA, 1% Triton X-100, 2.5 mmol/L sodium pyrophosphate, 1 mmol/L β-glycerolphosphate, 1 mmol/L Na_3_VO_4_, 1 μg/mL leupeptin, 1 mmol/L phenylmethylsufonylfluoride) for 30 min on ice. Lysates were cleared by centrifugation in an Eppendorff tube (15 min at 14 000 × g, 4°C). Protein content was determined against a standardized control using the Pierce Protein kit (Rockford, IL). Equal amounts of protein were diluted in 5× Laemmli sample buffer, heated for 10 min at 70°C, subjected to electrophoresis on 3–8% NuPAGE Tris-Acetate gels and transferred to nitrocellulose membranes. Non-specific binding was minimized by blocking for 1 h at room temperature with TBS-T (25 mM Tris-HCl pH 7.5, 150 mM NaCl and 0.05% Tween-20) containing 5% (w/v) non-fat dry milk. Filters were washed in TBS-T and incubated with primary antibodies for 2 h at room temperature in TBS-T containing 5% (w/v) non-fat dry milk. Membranes were washed in TBS-T, horseradish peroxidase-conjugated secondary antibodies in TBS-T were added for 45 min, and immunoreactive bands were detected by the enhanced chemiluminiscence reagent (Pierce, Rockford, IL). Blots were re-probed with an antibody for β-actin. Densitometric values of protein bands were quantified using Scion Imaging Software (Scion Corp., Frederick, MD).

### *In situ* immunofluorescent staining of FASN

Cells were seeded at 1 × 10^4^ cells/well in a fourwell chamber slide (Nalge Nunc International, Rochester, NY). After 48 h incubation with graded concentrations of U0126 or LY294002, cells were washed with PBS, fixed in 4% paraformaldehyde in PBS for 10 min, permeabilized with 0.2% Triton X-100/PBS for 15 min, and stored overnight at 4°C with 10% horse serum in PBS. The cells were washed and then incubated for 2 h with an anti- FASN mouse monoclonal antibody diluted 1:200 in 0.05% Triton X-100/PBS. After extensive washes, the cells were incubated for 45 min with FITC-conjugated anti-mouse IgG secondary antibody (Jackson ImmunoResearch Labs, West Grove, PA) diluted 1:200 in 0.05% Triton X-100/PBS. The cells were washed five times with PBS and mounted with VECTASHIELD + DAPI (Vector Laboratories, Burlingame, CA). As controls, cells were stained with primary or secondary antibody alone. No significant fluorescence was found in control experiments (data not shown). Indirect immunofluorescence was recorded on a Zeiss microscope. Images were noisefiltered, corrected for background, and prepared using Adobe Photoshop.

### Soft agar colony formation assays

Cells were grown in phenol red-free IMEM and 5% CCS for 5 days in T-75 flasks to deplete E_2_. A bottom layer of 1 mL IMEM containing 0.6% agar and 10% CCS was prepared in 35-mm multi-well cluster dishes. After the bottom layer solidified, cells (20,000/dish) were added in a 1 ml top layer containing either E_2_, ICI-182,780, cerulenin, C75, combinations thereof, ethanol (*v/v*), or DMSO (*v/v*), and 10% CCS. Dishes were incubated in a humidified 5% CO_2_ incubator at 37°C, and colonies measuring ≥ 50 μm were counted after ~ 14 days using a cell colony counter after staining with nitroblue tetrazolium (Sigma). Assays were carried out in triplicate.

### ELISA of secreted VEGF

Cell lines were seeded in 100-mm plates and cultured in complete growth medium. After reaching ~75 % confluence, the cells were washed twice with pre-warmed PBS and cultured in serum-free medium overnight. Cells were cultured in 0.1 % FBS-IMEM at 37°C for up to 48 h. After this time, the conditioned medium was aspirated, cleared by centrifugation at 1100 × *g* for 10 min at 4°C, and stored at −80°C until analysis. VEGF protein levels in conditioned media were determined by a VEGF ELISA (R & D Systems, Minneapolis, MN), as per the manufacturer's instructions.

### Cellular viability assessment using propidium iodide dye

Propidium iodide (PI) is a membrane impermeant DNA dye that is generally excluded from viable cells. After treatment with C75 (48 h), the cells were washed in cold PBS + 1% FBS and incubated with RNAse A (0.2 mg/mL) in PBS at 37°C for 20 min. PI was added to the cell suspension to reach a concentration of 20 μg/mL in PBS. The cells were then incubated for a further 30 min in the darkness at room temperature. The cells were then analyzed in a FACScalibur flow cytometer (Becton Dickinson, San Jose, CA). CellQuest software (Becton Dickinson) was run for data acquisition and analysis.

### Statistical analysis

All observations were confirmed by at least three independent experiments. Data are presented as means ± S.D. Student's *t* test (paired and unpaired) was used to evaluate the statistical significance of mean values (two tailed). Statistical significance level was P < 0.005 (denoted as *).
